# Nonlinear Static Bending and Forced Vibrations of Single-Layer MoS_2_ with Thermal Stress

**DOI:** 10.3390/ma17081735

**Published:** 2024-04-10

**Authors:** Xiaolin Chen, Kun Huang, Yunbo Zhang

**Affiliations:** Department of Engineering Mechanics, Faculty of Civil Engineering and Mechanics, Kunming University of Science and Technology, Kunming 650500, China; 20212110020@stu.kust.edu.cn (X.C.); yunbozhang@stu.kust.edu.cn (Y.Z.)

**Keywords:** single-layer MoS_2_, Galerkin method, multiscale method, thermal stress, 1:3 internal resonance

## Abstract

Single-layer molybdenum disulfide (MoS_2_) has been a research focus in recent years owing to its extensive potential applications. However, how to model the mechanical properties of MoS_2_ is an open question. In this study, we investigate the nonlinear static bending and forced vibrations of MoS_2_, subjected to boundary axial and thermal stresses using modified plate theory with independent in-plane and out-of-plane stiffnesses. First, two nonlinear ordinary differential equations are obtained using the Galerkin method to represent the nonlinear vibrations of the first two symmetrical modes. Second, we analyze nonlinear static bending by neglecting the inertial and damping terms of the two equations. Finally, we explore nonlinear forced vibrations using the method of multiple scales for the first- and third-order modes, and their 1:3 internal resonance. The main results are as follows: (1) The thermal stress and the axial compressive stress reduce the MoS_2_ stiffness significantly. (2) The bifurcation points of the load at the low-frequency primary resonance are much smaller than those at high frequency under single-mode vibrations. (3) Temperature has a more remarkable influence on the higher-order mode than the lower-order mode under the 1:3 internal resonance.

## 1. Introduction

Since monolayer graphene was first mechanically exfoliated from graphite in 2004 [1], its excellent physical, chemical, and mechanical properties have attracted extensive attention [2,3,4,5,6,7]. At the same time, the graphene-like two-dimensional (2D) transition metal dichalcogenides (TMDCs) have attracted widespread attention due to their single-layer characteristics and their excellent mechanical properties similar to those of graphene [8,9,10,11,12,13]. Molybdenum disulfide (MoS_2_) is a typical TMDC material, it can be obtained using mechanical stripping, a chemical approach, CVD synthesis, and other methods [14,15,16]. There are significant differences in the size, quality, and yield of monolayer molybdenum disulfide prepared using different methods. MoS_2_ not only overcomes the zero-band-gap drawback of graphene but also retains its numerous advantages. This makes it suitable for a broad range of potential applications [15,17,18]. Thus far, research on MoS_2_ has focused on its electrical, thermal, and friction properties [19], whereas its mechanical properties have rarely been investigated. TMDCs have been used as high-quality nanoresonators [20,21]. Because the band structure of monolayer MoS_2_ can be changed according to the mechanical strain, new nanomechanical devices can be designed by applying mechanical deformation. For example, Andres [22] fabricated a single-layered mechanical resonator using MoS_2_ and demonstrated nonlinear behavior under room temperature and vacuum conditions. However, how to model the mechanical properties of 2D nanomaterials, in which the materials have a monolayer structure with single or multiple atoms, remains an open question. Because monolayer MoS_2_ can resist bending deformations, the macroscopic Föppl-von Karman plate theory has been used by most researchers to model its mechanical properties. The deformation energy density of the classical Föppl-von Karman plate is as follows [23]:

(1) $$U=\frac{1}{2}\int_{s}\left\{D\left[{\left(2H\right)}^{2}+2\left(1-\nu \right)K\right]+D_{1}\left[{\left(2J\right)}^{2}-2\left(1-\nu \right)Q\right]\right\}\;d\;S,$$

where $D=\left[Eh^{3}/12\left(1-\nu^{2}\right)\right]$ is the bending stiffness; $D_{1}=Eh/\left(1-\nu^{2}\right)$ is the extensional stiffness; and E, ν, and h are the elastic modulus, Poisson’s ratio, and thickness of the plate, respectively. This indicates that the bending and extensional stiffnesses are in $D/D_{1}=h^{2}/12$ for classical plate theory, whereas the two stiffnesses in 2D monolayer nanomaterials are independent, namely $D/D_{1}\neq h^{2}/12$ [23]. Therefore, one cannot obtain the out-of-plane bending and torsional stiffnesses using the in-plane mechanical parameters and the thickness of the 2D monolayer materials. This is called the Yakobson paradox [23], whereas some authors believe that there is no paradox because much of the literature fails to distinguish h and E from the effective thickness and the effective elastic modulus [24]. Similarly, for a single-layer MoS_2_, one cannot directly obtain the out-of-plane bending and the torsional stiffness through its thickness. Based on the bond orbital theory of covalent bonds, Huang [25] obtained a continuous mechanical theory of monolayer graphene to explain the Yakobson paradox physically. This theory clarifies the physical mechanism of graphene resistance to deformations. The theory proves that graphene has two independent in-plane mechanical parameters and two independent out-of-plane mechanical parameters. Subsequently, Huang et al. obtained the deformation energy density of hexagonal boron nitride (h-BN) using the DREIDING force field, and also proved that the monolayer h-BN has four independent mechanical parameters [26]. By combining the classical fracture theory and the interaction potential of carbon atoms, the researchers in [27] theoretically explained the brittle fracture of graphene. The above studies demonstrate that the macroscopic continuum mechanics theory needs modification to describe the mechanical behaviors of nanomaterials.

The existing MoS_2_ molecular dynamics (MD) calculations have shown that the bending stiffness obtained using classical plate theory with the thickness of the three layers of atoms (h=3.2 nm) is not identical to the stiffness obtained using MD calculations [28]. To solve this contradiction, Huang proposed a nonlinear plate theory with independent in-plane and out-of-plane mechanical parameters to model MoS_2_ mechanical behaviors based on finite temperature [29]. This theory has a deformation energy density similar to classical plate theory, but it has four independent mechanical parameters. This new theory abandons the equivalent thickness of MoS_2_ and directly takes in-plane and out-of-plane stiffnesses as independent mechanical parameters. Consequently, the Yakobson paradox is effectively avoided.

Two-dimensional nanomaterials are typically sensitive to temperature because their out-of-plane stiffness is low. MoS_2_ expands with increasing temperature [30,31]. Recent MD calculations have shown that temperature changes have little influence on the elastic parameters of MoS_2_; however, temperature can cause significant thermal expansion [32]. For single-layer MoS_2_ with immovable boundaries, thermal expansion may induce thermal stress, which can lead to thermal buckling. In this study, the nonlinear static bending and vibrations of single-layer MoS_2_ with four hinged edges were investigated based on a modified plate model proposed by Huang [29]. This study focuses on the influence of temperature on the nonlinear mechanical behavior of monolayer MoS_2_.

## 2. Materials and Methods

A modified Föppl-von Karman plate model with independent in-plane and out-of-plane stiffnesses was established by Huang to model the mechanical properties of single-layer MoS_2_ [29]. Because Huang’s theory was published in Chinese, we briefly review this new theory for reader understanding.

**Figure 1:**
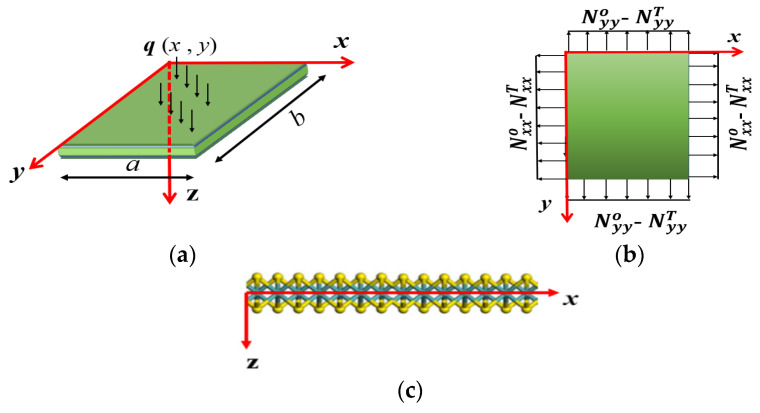
Calculation diagram of a single-layer MoS_2_ under load: (**a**) plate model with the coordinate; (**b**) applied edge loads; (**c**) side view of the MoS_2_ lattice structure.

Single-layer MoS_2_ is considered a 2D plate in Huang’s theory, as shown in Figure 1, and its deformation energy density is as follows [29]:

(2) $$U=\int_{s}\left[\frac{1}{2}k_{B}{\left(2H\right)}^{2}+k_{G}K+\frac{1}{2}k_{b}{\left(2J\right)}^{2}-k_{g}Q\right]dS.$$

Here, H and K are the mean and Gaussian curvatures, respectively, of the deformed MoS_2_ middle surface. $Q=\det\left(\varepsilon_{ij}^{0}\right)$ and $J=\mathrm{tr}\left(\varepsilon_{ij}\right)$ are the two invariants of the 2D strain tensor $\varepsilon_{ij}^{0},\;i,j=x,y$ on the middle surface. $k_{B}$ and $k_{G}$ are independent bending stiffness and torsional stiffness (Gaussian stiffness), respectively, whereas $k_{b}$ and $k_{g}$ are in-plane stiffness parameters. These four independent stiffness parameters are obtained through atomic simulations and experiments.

Using the von Karman nonlinear strain, the components of the strain tensor can be expressed as follows:

(3) $$\begin{array}{l} \varepsilon_{xx}^{0}=\frac{\partial u}{\partial x}+\frac{1}{2}{\left(\frac{\partial w}{\partial x}\right)}^{2},\;\varepsilon_{yy}^{0}=\frac{\partial v}{\partial y}+\frac{1}{2}{\left(\frac{\partial w}{\partial y}\right)}^{2},\\ \varepsilon_{xy}^{0}=\frac{1}{2}\left(\frac{\partial u}{\partial y}+\frac{\partial v}{\partial x}+\frac{\partial w}{\partial x}\frac{\partial w}{\partial y}\right). \end{array}$$

Here, u, v, and w are the displacements of the middle surface in the x, y, and z directions, respectively. Equation (3) is consistent with the classical plate in addition to the four stiffness parameters. Therefore, we can define the Airy function of the in-plane 2D stress as $N_{xx}=\partial^{2}F/\partial y^{2}$ and $N_{yy}=\partial^{2}F/\partial x^{2}$. From Equation (2), we obtain

(4) $$\begin{array}{l} N_{xx}=k_{b}\varepsilon_{xx}^{0}+\left(k_{b}-k_{g}\right)\varepsilon_{yy}^{0},\;N_{xy}=k_{g}\varepsilon_{xy}^{0},\\ N_{yy}=\left(k_{b}-k_{g}\right)\varepsilon_{xx}^{0}+k_{b}\varepsilon_{yy}^{0}. \end{array}$$

Therefore, the in-plane strain is expressed as follows:

(5) $$\begin{array}{l} \varepsilon_{xy}^{0}=\frac{N_{xy}}{k_{g}},\;\varepsilon_{xx}^{0}=\frac{1}{\chi }N_{xx}-\frac{1}{\lambda }N_{yy},\\ \varepsilon_{yy}^{0}=\frac{1}{\lambda }N_{xx}-\frac{1}{\chi }N_{yy}, \end{array}$$

where $\chi =k_{g}\left(2k_{b}-k_{g}\right)k_{b}^{-1}$ and $\lambda =k_{g}\left(2k_{b}-k_{g}\right){\left(k_{b}-k_{g}\right)}^{-1}$. According to Equation (5), the in-plane strain energy density can be rewritten as follows:

(6) $$U_{s}=\int_{S}\left\{\frac{1}{2\chi }{\left(\frac{\partial^{2}F}{\partial y^{2}}+\frac{\partial^{2}F}{\partial x^{2}}\right)}^{2}-\frac{1}{k_{g}}\left[\frac{\partial^{2}F}{\partial y^{2}}\frac{\partial^{2}F}{\partial x^{2}}-{\left(\frac{\partial^{2}F}{\partial x\partial y}\right)}^{2}\right]\right\}dS.$$

To ensure the continuity and single-value of the displacement field, the strain field must satisfy the following completeness condition [33]: $2\partial^{2}\varepsilon_{xy}^{0}/\partial x\partial y-\partial^{2}\varepsilon_{xx}^{0}/\partial y^{2}-\partial^{2}\varepsilon_{yy}^{0}/\partial x^{2}=K$. This equation can be rewritten as $\Delta^{2}F=-\chi K$. The Lagrange multiplier $l\left(x,y\right)$ must be introduced into the potential energy function because the stress function is introduced. Then, Equation (2) can be rewritten as follows:

(7) $$\begin{array}{l} U=\int_{s}\left\{\frac{1}{2}k_{B}{\left(2H\right)}^{2}+k_{G}K+\frac{1}{2\chi }{\left(\frac{\partial^{2}F}{\partial y^{2}}+\frac{\partial^{2}F}{\partial x^{2}}\right)}^{2}\right.-\\ \left.\frac{1}{k_{g}}\left[\frac{\partial^{2}F}{\partial y^{2}}\frac{\partial^{2}F}{\partial x^{2}}-{\left(\frac{\partial^{2}F}{\partial x\partial y}\right)}^{2}\right]+l\left(\Delta^{2}F+\chi K\right)\right\}dS. \end{array}$$

By performing complex but direct computations on Equation (7) and identifying the Lagrange multiplier, Equation (7) can be transformed into

(8) $$\begin{array}{l} U=\int_{s}\left\{\frac{1}{2}k_{B}{\left(2H\right)}^{2}+k_{G}K+\frac{1}{2\chi }{\left(\frac{\partial^{2}F}{\partial y^{2}}+\frac{\partial^{2}F}{\partial x^{2}}\right)}^{2}\right.-\frac{1}{k_{g}}\left[\frac{\partial^{2}F}{\partial y^{2}}\frac{\partial^{2}F}{\partial x^{2}}-{\left(\frac{\partial^{2}F}{\partial x\partial y}\right)}^{2}\right]\\ +\frac{1}{2}\left[\frac{\partial^{2}F}{\partial y^{2}}{\left(\frac{\partial w}{\partial x}\right)}^{2}\right.\left.\left.+\frac{\partial^{2}F}{\partial x^{2}}{\left(\frac{\partial w}{\partial y}\right)}^{2}-2\frac{\partial^{2}F}{\partial x\partial y}\frac{\partial w}{\partial x}\frac{\partial w}{\partial y}\right]\right\}dS. \end{array}$$

Considering the influence of temperature on MoS_2_, Huang applied a boundary axial external force and thermal stress to the structure [29]; therefore, the load work is as follows:

(9) $$W=\int_{S}\left\{q\left(x,y,t\right)w+\frac{1}{2}\left[\left(N_{xx}^{0}-N_{xx}^{T}\right){\left(\frac{\partial w}{\partial x}\right)}^{2}\right.\right.\left.\left.+2N_{xy}^{0}\frac{\partial w}{\partial x}\frac{\partial w}{\partial y}+\left(N_{yy}^{0}-N_{yy}^{T}\right){\left(\frac{\partial w}{\partial y}\right)}^{2}\right]\right\}dS,$$

where $q\left(x,y,t\right)$ is the load in the z direction, and $N_{xx}^{0}$ and $N_{yy}^{0}$ are the pre-applied axial tensile stresses in the *x* and *y* directions at the boundaries, respectively. $N_{xx}^{T}$ and $N_{yy}^{T}$ are the thermal stresses in the *x* and *y* directions at the boundaries [29,34]. The thermal stresses in the uniform temperature field are $N_{xx}^{T}=k_{b}\varepsilon_{xx}^{T}=k_{b}\alpha Τ$ and $N_{yy}^{T}=k_{g}\varepsilon_{yy}^{T}=k_{g}\alpha Τ$, where $\varepsilon_{xx}^{T}$ and $\varepsilon_{yy}^{T}$ represent the thermal strain, and α is the coefficient of thermal expansion (CTE).

The Lagrange function can be constructed as L=U−W, which is subjected to variational calculations, namely, letting δL=0 with the independent variables w and F. Therefore, we obtain the force balance equation and compatibility condition as follows:

(10) $$\left\{\begin{array}{l} K_{B}\nabla^{4}w=\left(\frac{\partial^{2}F}{\partial y^{2}}+N_{xx}^{0}-N_{xx}^{T}\right)\frac{\partial^{2}w}{\partial x^{2}}+\left(\frac{\partial^{2}F}{\partial x^{2}}+N_{yy}^{0}\right.\left.-N_{yy}^{T}\right)\frac{\partial^{2}w}{\partial y^{2}}-2\left(\frac{\partial^{2}F}{\partial x\partial y}+N_{xy}^{0}\right)\frac{\partial^{2}w}{\partial x\partial y}+q,\\ \nabla^{4}F=-\chi \left[\frac{\partial^{2}w}{\partial x^{2}}\frac{\partial^{2}w}{\partial y^{2}}-{\left(\frac{\partial^{2}w}{\partial x\partial y}\right)}^{2}\right]. \end{array}\right.$$

Equation (10) is a mathematical model of MoS_2_ derived by Huang [29], in which the out-of-plane and in-plane stiffness parameters are independent. To study the dynamics problem, we add the inertial force term $m\left(\partial^{2}w/\partial t^{2}\right)$ to Equation (10) using the D’Alembert principle [35]; thus, the move equations can be rewritten as follows:

(11) $$\begin{array}{l} K_{B}\nabla^{4}w=\left(\frac{\partial^{2}F}{\partial y^{2}}+N_{xx}^{0}-N_{xx}^{T}\right)\frac{\partial^{2}w}{\partial x^{2}}+\left(\frac{\partial^{2}F}{\partial x^{2}}+N_{yy}^{0}-N_{yy}^{T}\right)\frac{\partial^{2}w}{\partial y^{2}}\\ -2\left(\frac{\partial^{2}F}{\partial x\partial y}+N_{xy}^{0}\right)\frac{\partial^{2}w}{\partial x\partial y}+q\left(x,y,t\right)-m\frac{\partial^{2}w}{\partial t^{2}}, \end{array}$$

(12) $$\nabla^{4}F=-\chi \left[\frac{\partial^{2}w}{\partial x^{2}}\frac{\partial^{2}w}{\partial y^{2}}-{\left(\frac{\partial^{2}w}{\partial x\partial y}\right)}^{2}\right].$$

For simplification, we assume that $q\left(x,y,t\right)$ in Equation (11) is a harmonic load; therefore,

(13) $$q\left(x,y,t\right)=f\left(x,y\right)\cos\left(\Omega t\right).$$

Here, we define the following dimensionless variables as follows:

(14) $$\begin{array}{l} \tilde{w}=\frac{w}{a},\;\tilde{x}=\frac{x}{a},\;\tilde{y}=\frac{y}{b},\;\tilde{t}=\omega_{0}t,\\ \tilde{F}=\frac{F}{K_{B}},\;\tilde{\Omega }=\frac{\Omega }{\omega_{0}},\;\omega_{0}=\sqrt{\frac{K_{B}\pi^{4}}{ma^{4}}}, \end{array}$$

where a and b represent the side lengths of the monolayer MoS_2_, as shown in Figure 1. Thus, Equation (11) can be simplified into a dimensionless form as follows:

(15) $$\frac{\partial^{2}\tilde{w}}{\partial {\tilde{t}}^{2}}+\frac{1}{\pi^{4}}\frac{\partial {\tilde{w}}^{4}}{\partial {\tilde{x}}^{4}}+\frac{2a^{2}}{\pi^{4}b^{2}}\frac{\partial {\tilde{w}}^{4}}{\partial {\tilde{x}}^{2}{\tilde{y}}^{2}}+\frac{a^{4}}{\pi^{4}b^{4}}\frac{\partial {\tilde{w}}^{4}}{\partial {\tilde{y}}^{4}}=\frac{a^{2}}{\pi^{4}b^{2}}\frac{\partial^{2}\tilde{F}}{\partial {\tilde{y}}^{2}}\frac{\partial^{2}\tilde{w}}{\partial {\tilde{x}}^{2}}\;\begin{array}{c} +\frac{\left(N_{xx}^{0}-N_{xx}^{T}\right)}{ma^{2}\omega_{0}^{2}}\frac{\partial^{2}\tilde{w}}{\partial {\tilde{x}}^{2}}+\frac{a^{2}}{\pi^{4}b^{2}}\frac{\partial^{2}\tilde{F}}{\partial {\tilde{x}}^{2}}\frac{\partial^{2}\tilde{w}}{\partial {\tilde{y}}^{2}}+\frac{N_{yy}^{0}-N_{yy}^{T}}{mb^{2}\omega_{0}^{2}}\frac{\partial^{2}\tilde{w}}{\partial {\tilde{y}}^{2}}\\ -2\frac{a^{2}}{\pi^{4}b^{2}}\frac{\partial^{2}\tilde{F}}{\partial \tilde{x}\partial \tilde{y}}\frac{\partial^{2}\tilde{w}}{\partial \tilde{x}\partial \tilde{y}}-2\frac{N_{xy}^{0}}{mab\omega_{0}^{2}}\frac{\partial^{2}\tilde{w}}{\partial \tilde{x}\partial \tilde{y}}+\frac{a^{3}f}{\pi^{4}K_{B}}\cos\tilde{\Omega }\tilde{t}, \end{array}$$

(16) $$\frac{b^{2}K_{B}}{a^{4}}\frac{\partial^{4}\tilde{F}}{\partial {\tilde{x}}^{4}}+\frac{2K_{B}}{a^{2}}\frac{\partial^{4}\tilde{F}}{\partial {\tilde{x}}^{2}{\tilde{y}}^{2}}+\frac{K_{B}}{b^{2}}\frac{\partial^{4}\tilde{F}}{\partial {\tilde{y}}^{4}}=-\chi \left[\frac{\partial^{2}\tilde{w}}{\partial {\tilde{x}}^{2}}\frac{\partial^{2}\tilde{w}}{\partial {\tilde{y}}^{2}}-{\left(\frac{\partial^{2}\tilde{w}}{\partial \tilde{x}\partial \tilde{y}}\right)}^{2}\right].$$

### 2.1. Analysis of Static Bending

Because Equations (15) and (16) are nonlinear partial differential equations, they are difficult to solve accurately. Therefore, the Galerkin method [36] is used to transform Equations (15) and (16) into ordinary differential equations in time. Equations (15) and (16) resemble the classical plate (but the mechanical parameters of the MoS_2_ are independent). Under small deformations, symmetric loads may only induce symmetric deformations, even when nonlinear terms emerge. We then analyze the static and dynamic bending deformations under symmetric loads through first- and third-order symmetric modes. We expand the transverse displacement w and stress function F as follows:

(17) $$\left\{\begin{array}{l} \tilde{w}=u_{1}\left(\tilde{t}\right)\sin\pi \tilde{x}\sin\pi \tilde{y}+u_{3}\left(\tilde{t}\right)\sin3\pi \tilde{x}\sin\pi \tilde{y},\\ \tilde{F}={\tilde{\xi }}_{11}\left(\tilde{t}\right)\sin\pi \tilde{x}\sin\pi \tilde{y}+{\tilde{\xi }}_{31}\left(\tilde{t}\right)\sin3\pi \tilde{x}\sin\pi \tilde{y}. \end{array}\right.$$

Substituting the $\tilde{F}$ in Equation (17) into Equation (16) and multiplying $\sin\pi \tilde{x}\sin\pi \tilde{y}$ and $\sin3\pi \tilde{x}\sin\pi \tilde{y}$ on the two sides of Equation (16) (the Galerkin model), we have

(18) $$\left\{\begin{array}{l} \xi_{11}=k_{1}^{*}\eta_{11}^{2}+k_{2}^{*}\eta_{11}\eta_{31}+k_{3}^{*}\eta_{31}^{2},\\ \xi_{31}=k_{4}^{*}\eta_{11}^{2}+k_{5}^{*}\eta_{11}\eta_{31}+k_{6}^{*}\eta_{31}^{2}. \end{array}\right.$$

The parameters in Equation (18) are as follows:

(19) $$\begin{array}{l} k_{1}^{*}=\frac{-16a^{4}b^{2}\chi }{3\pi^{2}K_{B}{\left(a^{2}+b^{2}\right)}^{2}},\;k_{2}^{*}=\frac{352a^{4}b^{2}\chi }{45\pi^{2}K_{B}{\left(a^{2}+b^{2}\right)}^{2}},k_{3}^{*}=\frac{-912a^{4}b^{2}\chi }{35\pi^{2}K_{B}{\left(a^{2}+b^{2}\right)}^{2}},\;\\ k_{4}^{*}=\frac{176a^{4}b^{2}\chi }{45\pi^{2}K_{B}{\left(a^{2}+9b^{2}\right)}^{2}},k_{5}^{*}=\frac{-1824a^{4}b^{2}\chi }{35\pi^{2}K_{B}{\left(a^{2}+9b^{2}\right)}^{2}},\;k_{6}^{*}=\frac{-16a^{4}b^{2}\chi }{\pi^{2}K_{B}{\left(a^{2}+9b^{2}\right)}^{2}}. \end{array}$$

Similarly, we substitute the $\tilde{w}$ in Equation (17) into Equation (15); subsequently, we multiply $\sin\pi \tilde{x}\sin\pi \tilde{y}$ and $\sin3\pi \tilde{x}\sin\pi \tilde{y}$ on the two sides of Equation (15), and considering Equation (18), the vibration equations for the first-order and third-order modes can be obtained as follows:

(20) $$\left\{\begin{array}{l} {\ddot{u}}_{1}+\omega_{1}^{2}u_{1}=\alpha_{1}u_{1}^{3}+\alpha_{2}u_{1}^{2}u_{3}+\alpha_{3}u_{1}u_{3}^{2}+\alpha_{4}u_{3}^{3}+f_{1}\cos\Omega t,\\ {\ddot{u}}_{3}+\omega_{3}^{2}u_{3}=\alpha_{5}u_{1}^{3}+\alpha_{6}u_{1}^{2}u_{3}+\alpha_{7}u_{1}u_{3}^{2}+\alpha_{8}u_{3}^{3}+f_{3}\cos\Omega t. \end{array}\right.$$

The parameters in Equation (20) are listed in Appendix A. By omitting the inertial terms in Equation (20), the static deformations of the midpoint of the monolayer MoS_2_ are obtained as follows:

(21) $$\left\{\begin{array}{l} \omega_{1}^{2}u_{1}=\alpha_{1}u_{1}^{3}+\alpha_{2}u_{1}^{2}u_{3}+\alpha_{3}u_{1}u_{3}^{2}+\alpha_{4}u_{3}^{3}+f_{1},\\ \omega_{3}^{2}u_{3}=\alpha_{5}u_{1}^{3}+\alpha_{6}u_{1}^{2}u_{3}+\alpha_{7}u_{1}u_{3}^{2}+\alpha_{8}u_{3}^{3}+f_{3}. \end{array}\right.$$

**Figure 2:**
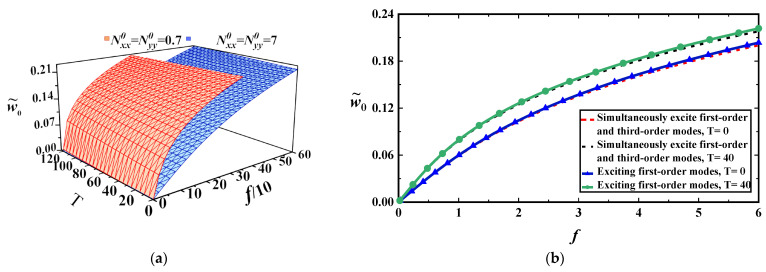
(**a**) Static deformation amplitudes (${\tilde{w}}_{0}=\tilde{w}\left(0.5,0.5\right)$) under the loads and temperature for a=b=6 nm; (**b**) static deformation amplitudes with the loads for two temperatures at a=5 nm, b=10 nm for $N_{xx}^{0}=0.3\;\mathrm{nN}/\mathrm{nm}$, $N_{yy}^{0}=0.1\;\mathrm{nN}/\mathrm{nm}$.

We take the MoS_2_ mechanical parameters in Table 1 as an example. Using the data in the table and Equation (21), the static bending amplitudes under external loads are obtained with four hinged edges, as shown in Figure 2a,b.

The two figures show that the thermal stress and axial compressive stress decrease the MoS_2_ stiffness. If both the first- and third-order modes are considered, the deflections of the midpoint would be slightly smaller than those considering only the first-order mode, as shown in Figure 2b. This may indicate that the first-order mode can precisely represent static deformations under symmetric loads.

### 2.2. Nonlinear Primary Resonance without Internal Resonance

In this section, our study of the nonlinear vibrations of single-layer MoS_2_ using Equation (20) are presented. These equations only contain cubic nonlinear terms. If the geometric dimensions of MoS_2_ and the axial force have the given values, the equations may exhibit a 1:3 internal resonance. This study mainly includes the following three parts: exciting only the first- or third-order primary resonance and the 1:3 internal resonance with the load’s frequency near the low-order natural frequency.

To simplify the analysis, we assume that the damping force is $2{\hat{c}}_{j}{\dot{u}}_{j}$ with damping coefficient ${\hat{c}}_{j}$. If there is no internal resonance in Equation (20), the vibrations of the unexcited modes rapidly decay because of damping. Therefore, the steady-state vibrations only contain the excited mode [39]. Thus, the coupling terms in Equation (20) can be neglected. The forced vibration equation of single-layer MoS_2_ in the first-order mode ($\Omega \approx \omega_{1}$) or third-order mode ($\Omega \approx \omega_{3}$) is as follows:

(22) $${\ddot{u}}_{j}+\omega_{j}^{2}u_{j}+2{\hat{c}}_{j}{\dot{u}}_{j}=\beta_{j}u_{j}^{3}+f_{j}\cos\left(\Omega t\right),j=1\;\mathrm{or}\;3,$$

where $\beta_{1}=\alpha_{1},\;\beta_{3}=\alpha_{8}$. We employ the multiple scale perturbation method to analyze Equation (22). The method is a classical perturbation method that is used to solve weak nonlinear differential equations. We supposed that the influences of damping, the nonlinear terms, and the loads emerge in a unified perturbation equation, so we set ${\hat{c}}_{j}=\varepsilon^{2}c_{j}$ and $f_{j}=\varepsilon^{3}f_{j1}$. The small parameter ε=0.1 is used in this research. Consequently, Equation (22) is rewritten as follows:

(23) $${\ddot{u}}_{j}+\omega_{j}^{2}u_{j}+2\varepsilon^{2}c_{j}{\dot{u}}_{j}=\beta_{j}u_{j}^{3}+\varepsilon^{3}f_{j1}\cos\left(\Omega t\right),j=1\;\mathrm{or}\;3$$

We assume the solution of Equation (23) as

(24) $$u_{j}=\varepsilon u_{j1}\left({Τ}_{0},{Τ}_{2}\right)+\varepsilon^{3}u_{j3}\left({Τ}_{0},{Τ}_{2}\right)+\cdots ,$$

where ${Τ}_{0}=t$, ${Τ}_{1}=\varepsilon t$, and ${Τ}_{2}=\varepsilon^{2}t$. By substituting Equation (24) into Equation (23) and equating the coefficients of ε and $\varepsilon^{3}$ on both sides, we obtain

(25) $$\varepsilon :\;D{\;}_{0}^{2}u_{j1}+\omega {\;}_{j}^{2}u_{j1}=0,$$

(26) $$\varepsilon^{3}:\;D_{0}^{2}u_{j3}+\omega_{j}^{2}u_{j3}=-2D_{0}\left(D_{2}u_{j1}\right.\left.+c_{j}u_{j1}\right)+\beta_{j}u_{j1}^{3}+f_{j1}\cos\left(\Omega T_{0}\right),$$

where $D_{0}=d/dT_{0}$ and $D_{2}=d/dT_{2}$. In accordance with the ordinary differential equation theory, the solution of Equation (25) is as follows:

(27) $$u_{j1}=A_{j}\left(T_{2}\right)\exp\left(i\omega_{j}T_{0}\right)+cc,$$

where cc denotes the complex conjugate of the preceding term, and $A_{j}$ are the arbitrary functions of $T_{2}$. Substituting Equation (27) into (26), we obtain

(28) $$\begin{array}{l} D_{0}^{2}u_{j3}+\omega_{j}^{2}u_{j3}=\left[-2i\omega_{j}\left(D_{1}A_{j}+c_{j}A_{j}\right)\right.\left.+3\beta_{j}A_{j}^{2}\bar{A}\right]\\ \exp\left(i\omega_{j}T_{0}\right)+\beta_{j}A_{j}^{3}\exp\left(3i\omega_{j}T_{0}\right)+\frac{1}{2}f_{j1}\exp\left(i\Omega T_{0}\right)+cc, \end{array}$$

where $\bar{A}$ is the complex conjugate of A. Letting $\Omega =\omega_{j}+\varepsilon^{2}\sigma_{j}$ and applying the elimination condition for the secular terms in Equation (28), we obtain

(29) $$-2i\omega_{j}\left(D_{1}A_{j}+c_{j}A_{j}\right)+3\beta_{j}A_{j}^{2}{\bar{A}}_{j}+\frac{1}{2}f_{j1}\exp\left(i\sigma_{j}T_{2}\right)=0.$$

We introduce the polar forms $A_{j}=\left(1/2\right)\lambda_{j}\exp\left(i\theta_{j}\right)$ with $\lambda_{j}$ and $\theta_{j}$ in the real functions of $T_{2}$. Substituting the $A_{j}$ into Equation (28) and separating the real and imaginary parts of Equation (29), we obtain

(30) $$\left\{\begin{array}{l} D_{1}\lambda_{j}+c\lambda_{j}=\frac{f_{j1}}{2\omega_{j}}\sin\gamma_{j},\\ \lambda_{j}D_{1}\theta_{j}+\frac{3}{8\omega_{j}}\beta_{j}\lambda_{j}^{3}=\frac{-1}{2\omega_{j}}f_{j1}\cos\gamma_{j}, \end{array}\right.$$

where $\gamma_{j}=\sigma_{j}T_{2}-\theta_{j}$.

The steady-state motion will occur if $D_{1}\lambda_{j}=D_{1}\gamma_{j}=0$. This corresponds to the solution of the following equations:

(31) $$\left\{\begin{array}{l} c_{j}\lambda_{j}=\frac{1}{2\omega_{j}}f_{j1}\sin\gamma_{j},\\ \lambda_{j}\sigma_{j}+\frac{3\beta_{j}}{8\omega_{j}}\lambda_{j}^{3}=\frac{-1}{2\omega_{j}}f_{j1}\cos\gamma_{j}. \end{array}\right.$$

#### 2.2.1. Primary Resonance of Low Frequency without Internal Resonance

The vibration amplitude of the low-frequency primary resonance can be obtained from Equation (31) using j=1:

(32) $$\left[{c_{1}}^{2}+{\left(\sigma_{1}+\frac{3\beta_{1}}{8\omega_{1}}\lambda_{1}^{2}\right)}^{2}\right]\lambda_{1}^{2}=\frac{f_{11}^{2}}{4\omega_{1}^{2}}.$$

One can obtain $\lambda_{1}$ from Equation (32). Then, we substitute it into Equation (24), so the first-order approximate solution is obtained.

(33) $$u_{1}\approx \varepsilon \lambda_{1}\cos\left(\Omega t-\gamma_{1}\right).$$

The mechanical parameters of MoS_2_ are listed in Table 1. The geometric dimensions are a=5 nm and b=10 nm, and the axial tensile stresses are $N_{xx}^{0}=0.3\;\mathrm{nN}/\mathrm{nm}$ and $N_{yy}^{0}=0.1\;\mathrm{nN}/\mathrm{nm}$. These parameters prevent internal resonance. Because there has been no thorough research on damping, we use $2\varepsilon^{2}c_{j}=0.05$ for simplification. Therefore, we have $c_{j}=2.5,$ ${\hat{c}}_{j}=0.025$ for ε=0.1. By substituting these parameters into the expressions in Appendix A, we obtain $3\omega_{1}\ll \omega_{3}$.

**Figure 3:**
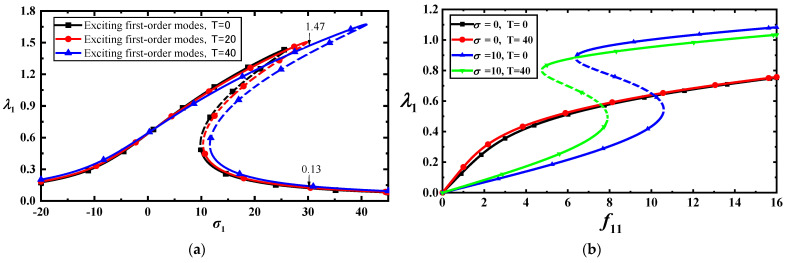
(**a**) Frequency–response curves of low-frequency primary resonance with the three temperatures for $f_{11}=10$; (**b**) load–response curves of vibration amplitudes with two temperatures.

**Figure 4:**
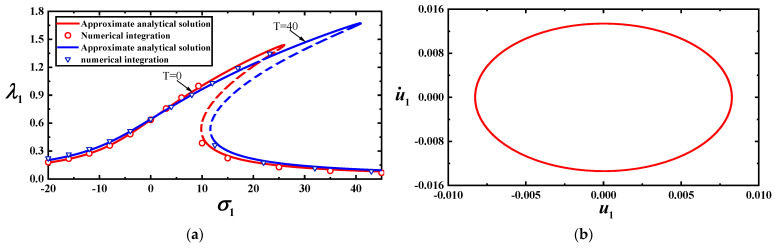
(**a**) Comparison between approximate analytical and numerical solutions for $f_{11}=10$; (**b**) phase diagram for $f_{11}=10,T=40$.

When these parameters are substituted into Equation (32), the amplitude–frequency response curves at three different temperatures were obtained with $f_{11}=10$, as shown in Figure 3a. This figure shows that temperature had an insignificant effect on the MoS_2_ amplitude–frequency response curve. However, the combination of the load frequency and temperature had a significant effect on the load–amplitude curve, as shown in Figure 3b. Figure 3a,b also shows that the amplitude–frequency and load–amplitude response curves have two bifurcation points that lead to jumps in the vibration amplitude. The dotted lines in Figure 3 and Figure 4 indicate unstable solutions. The stability of steady-state solutions can be determined through the eigenvalues of the Jacobian matrix of Equation (30); the details can be found in [39].

To validate the effectiveness of the approximate analytical solution, we simulate Equation (22) with $\left(f_{11},N_{xx}^{0},N_{yy}^{0}\right)=\left(10,0.3,0.1\right)$ using the Runge–Kutta method at T=0 and T=40, as shown in Figure 4a,b. Comparing the approximate analytical and numerical solutions, we find that the approximate analytical solution has good accuracy. The unstable solutions are indicated by dotted lines in Figure 3a,b and Figure 4a.

#### 2.2.2. Primary Resonance of High Frequency without Internal Resonance

For the primary high-frequency resonance, the vibration amplitude can be obtained from Equation (31) using j=3. We square the two equations and add them such that

(34) $$\left[c_{3}^{2}+{\left(\sigma_{3}+\frac{3\beta_{3}}{8\omega_{3}}\lambda_{3}^{2}\right)}^{2}\right]\lambda_{3}^{2}=\frac{f_{31}^{2}}{4\omega_{3}^{2}}.$$

Substituting the $\lambda_{3}$ and $\gamma_{3}$ determined by Equation (34) into Equation (24), we obtain the third-order approximate solution as follows:

(35) $$u_{3}\approx \varepsilon \lambda_{3}\cos\left(\Omega t-\gamma_{3}\right).$$

**Figure 5:**
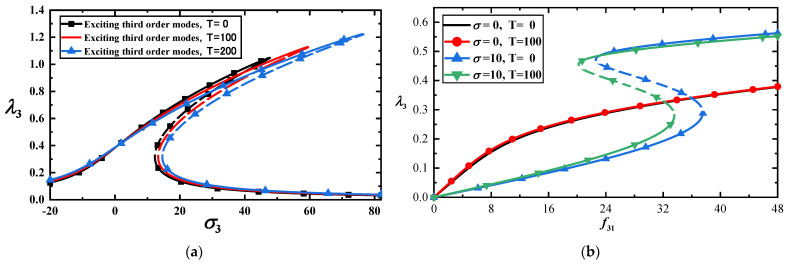
(**a**) Frequency–response curves of the high-frequency primary resonance with three temperatures for *f*_31_ = 50; (**b**) load–response curves of vibration amplitudes with two temperatures.

**Figure 6:**
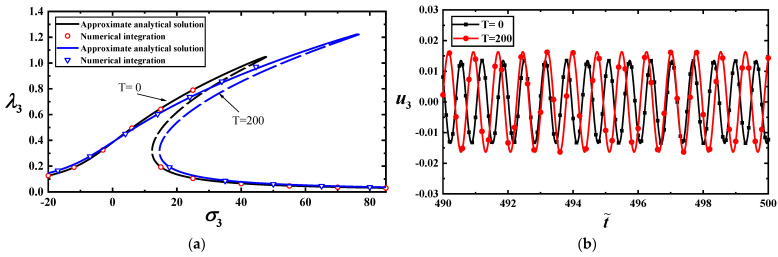
(**a**) Comparison between approximate analytical and numerical solutions with $f_{31}=50$ for T=0 or T=200; (**b**) time–response curves with $f_{31}=50,\sigma_{2}=20$ for T=0 or T=200.

The frequency–response or load–amplitude curves can be obtained using Equation (34) at different temperatures when the third-order mode is excited, as shown in Figure 5a,b. The precision of the approximate analytical solution is examined using the Runge–Kutta method as shown in Figure 6a,b. The dotted lines in Figure 5 and Figure 6 indicate the unstable solutions. The stability of the steady-state solutions can be determined using the eigenvalues of the Jacobian matrix of Equation (30); the details can be found in [39].

From Figure 5a,b and Figure 6a, the following three main results can be drawn. First, the vibration amplitudes of the third-order mode are significantly smaller than those of the first-order mode under the same load. Second, the temperature has little effect on the amplitude of the third-order mode. Finally, the bifurcation points of the $f_{11}$ of the low-frequency primary resonance are much smaller than those of the high-frequency resonance. A small bifurcation point of the first-order mode indicates that the low-frequency vibration is more prone to a large vibration amplitude. We use the Runge–Kutta method to calculate Equation (22) with $f_{31}=50$, as shown in Figure 6a,b. The unstable solutions are indicated by dotted lines in Figure 5a,b and Figure 6a.

**Figure 7:**
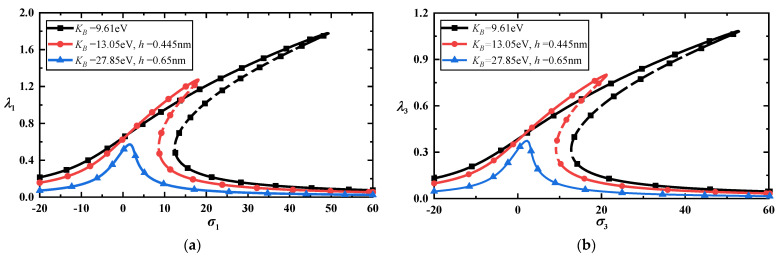
(**a**) Frequency–response curves of the low-frequency primary resonance with the classical plate model and the modified plate model” for f=10; (**b**) frequency–response curves of the high-frequency primary resonance with the classical plate model and the modified plate model for f=50.

Here, we have used two thicknesses [28], h=0.445 nm and h=0.65 nm, to show the differences in nonlinear vibrations between the classical Föppl-von Karman plate model and the modified Föppl-von Karman plate model in this paper. So, the partial mechanical parameters with different effective thicknesses are shown in Table 2. The outstanding differences in the frequency–response curves between the two models can be found from Figure 7a,b. 

#### 2.2.3. Primary Resonance and 1:3 Internal Resonance at Low Frequency

To research the 1:3 internal resonance, we rewrite Equation (20) as follows:

(36) $$\left\{\begin{array}{l} {\ddot{u}}_{1}+\omega_{1}^{2}u_{1}+2\varepsilon^{2}c_{1}{\dot{u}}_{1}=\alpha_{1}u_{1}^{3}+\alpha_{2}u_{1}^{2}u_{3}+\alpha_{3}u_{1}u_{3}^{2}\\ \;+\alpha_{4}u_{3}^{3}+\varepsilon^{3}f_{11}\cos\left(\Omega t\right),\\ {\ddot{u}}_{3}+\omega_{3}^{2}u_{3}+2\varepsilon^{2}c_{3}{\dot{u}}_{3}=\alpha_{5}u_{1}^{3}+\alpha_{6}u_{1}^{2}u_{3}+\alpha_{7}u_{1}u_{3}^{2}\\ \;+\alpha_{8}u_{3}^{3}+\varepsilon^{3}f_{31}\cos\left(\Omega t\right). \end{array}\right.$$

Here, we add two damping terms to the first- and third-order equations. The solution of Equation (36) are as follows:

(37) $$\left\{\begin{array}{l} u_{1}=\varepsilon u_{11}\left({Τ}_{0},{Τ}_{2}\right)+\varepsilon^{3}u_{13}\left({Τ}_{0},{Τ}_{2}\right)+\cdots ,\\ u_{3}=\varepsilon u_{31}\left({Τ}_{0},{Τ}_{2}\right)+\varepsilon^{3}u_{33}\left({Τ}_{0},{Τ}_{2}\right)+\cdots . \end{array}\right.$$

By substituting Equation (37) into Equation (36) and equating the coefficients of ε and $\varepsilon^{3}$, we obtain

(38) $$\varepsilon :\left\{\begin{array}{l} D_{0}^{2}u_{11}+\omega_{1}^{2}u_{11}=0,\\ D_{0}^{2}u_{31}+\omega_{3}^{2}u_{31}=0, \end{array}\right.$$

(39) $$\varepsilon^{3}:\left\{\begin{array}{l} D_{0}^{2}u_{13}+\omega_{1}^{2}u_{13}=-2D_{0}\left(D_{2}u_{11}+c_{1}u_{11}\right)+\alpha_{1}u_{11}^{3}+\alpha_{2}u_{11}^{2}u_{31}\\ \;+\alpha_{3}u_{11}u_{31}^{2}+\alpha_{4}u_{31}^{3}+f_{11}\cos\Omega T_{0},\\ D_{0}^{2}u_{33}+\omega_{3}^{2}u_{33}=-2D_{0}\left(D_{2}u_{31}+c_{3}u_{31}\right)+\alpha_{5}u_{11}^{3}+\alpha_{6}u_{11}^{2}u_{31}\\ \;+\alpha_{7}u_{11}u_{31}^{2}+\alpha_{8}u_{31}^{3}+f_{31}\cos\Omega T_{0}. \end{array}\right.$$

According to the theory of ordinary differential equations, we assume that the solution of Equation (38) are as follows:

(40) $$\left\{\begin{array}{l} u_{11}=A_{1}\left(T_{2}\right)\exp\left(i\omega_{1}T_{0}\right)+cc,\\ u_{31}=A_{2}\left(T_{2}\right)\exp\left(i\omega_{3}T_{0}\right)+cc, \end{array}\right.$$

Here, cc denotes the complex conjugate of the preceding terms, and $A_{1}$ $A_{2}$ represent the functions of $T_{2}$. Substituting Equation (40) into (39), we obtain

(41) $$\begin{array}{l} D_{0}^{2}u_{13}+\omega_{1}^{2}u_{13}=-\left[2i\omega_{1}\left(D_{2}A_{1}+c_{1}A_{1}\right)\right.+3\alpha_{1}A_{1}^{2}{\bar{A}}_{1}+\left.2\alpha_{3}A_{1}{\bar{A}}_{2}A_{2}\right]\exp\left(i\omega_{1}T_{0}\right)\\ +\alpha_{2}{\bar{A}}_{1}^{2}A_{2}\exp\left[i\left(-2\omega_{1}+\omega_{3}\right)T_{0}\right]+\frac{f_{31}}{2}\exp\left(i\Omega T_{0}\right)+cc+NST, \end{array}$$

(42) $$\begin{array}{l} D_{0}^{2}u_{33}+\omega_{2}^{2}u_{33}=-\left[2i\omega_{3}\left(D_{2}A_{2}+c_{3}A_{2}\right)\right.+3\alpha_{8}A_{2}^{2}{\bar{A}}_{2}+\left.2\alpha_{6}A_{1}{\bar{A}}_{1}A_{2}\right]\exp\left(i\omega_{3}T_{0}\right)\\ +\alpha_{5}A_{1}^{3}\exp\left(3i\omega_{1}T_{0}\right)+\frac{f_{31}}{2}\exp\left(i\Omega T_{0}\right)+cc+NST, \end{array}$$

where $\bar{A}$ denotes the complex conjugate of A and NST is the non-secular term.

These equations may exhibit an internal resonance of $\omega_{3}\approx 3\omega_{1}$. Introducing the detuning parameters $\sigma_{1}$ and $\sigma_{2}$, we obtain

(43) $$\omega_{3}=3\omega_{1}+\varepsilon^{2}\sigma_{\;1}.$$

(44) $$\Omega =\omega_{1}+\varepsilon^{2}\sigma_{2}.$$

Therefore, the solvability conditions of Equations (41) and (42) are as follows:

(45) $$\left\{\begin{array}{l} \frac{1}{2}f_{11}\exp\left(i\sigma_{1}T_{2}\right)-2i\omega_{1}\left(D_{2}A_{1}+c_{1}A_{1}\right)+3\alpha_{1}{A_{1}}^{2}{\bar{A}}_{1}\\ +2\alpha_{3}A_{2}{\bar{A}}_{2}A_{1}+\alpha_{2}{\bar{A}}_{1}^{2}A_{2}\exp\left(i\sigma_{1}T_{2}\right)=0,\\ -2i\omega_{3}\left(D_{2}A_{2}+c_{3}A_{2}\right)+\alpha_{5}A_{1}^{3}\exp\left(-i\sigma_{1}T_{2}\right)+2\alpha_{6}A_{1}{\bar{A}}_{1}A_{2}+3\alpha_{8}A_{2}^{2}{\bar{A}}_{2}=0. \end{array}\right.$$

If the polar coordinate form $A_{m}=\left(1/2\right)a_{m}\exp\left(i\beta_{m}\right),m=1,2$ is introduced and substituted into Equation (45), and the real and imaginary parts of the equation are separated, we obtain

(46) $$\left\{\begin{array}{l} 8\omega_{1}\left(D_{2}a_{1}+c_{1}a_{1}\right)=\alpha_{2}a_{1}^{2}a_{2}\sin\gamma_{1}+4f_{11}\sin\gamma_{2},\\ 8\omega_{3}\left(D_{2}a_{2}+c_{3}a_{2}\right)=-\alpha_{5}a_{1}^{3}\sin\gamma_{1},\\ 8\omega_{1}a_{1}D_{2}\beta_{1}=-\left(3\alpha_{1}a_{1}^{2}+2\alpha_{3}a_{2}^{2}\right)a_{1}-\alpha_{2}a_{1}^{2}a_{2}\cos\gamma_{1}-4f_{11}\cos\gamma_{2},\\ 8\omega_{3}a_{2}D_{2}\beta_{2}=-\left(3\alpha_{8}a_{2}^{2}+2\alpha_{6}a_{1}^{2}\right)a_{2}-\alpha_{5}a_{1}^{3}\cos\gamma_{1}. \end{array}\right.$$

Here, $\alpha_{m}$ and $\beta_{m}$ are real functions of $T_{2}$, and $\gamma_{1}=\sigma_{1}T_{2}-3\beta_{1}+\beta_{2}$, $\;\gamma_{2}=\sigma_{2}T_{2}-\beta_{1}$. The steady-state motion may occur when $D_{2}a_{m}=D_{2}\gamma_{m}=0$. The steady-state solution can be obtained using the following nonlinear equations:

(47) $$\left\{\begin{array}{l} 8\omega_{1}c_{1}a_{1}-\alpha_{2}a_{1}^{2}a_{2}\sin\gamma_{1}-4f_{11}\sin\gamma_{2}=0,\\ 8\omega_{3}c_{3}a_{2}+\alpha_{5}a_{1}^{3}\sin\gamma_{1}=0,\\ 8\omega_{1}a_{1}\sigma_{2}+\left(3\alpha_{1}a_{1}^{2}+2\alpha_{3}a_{2}^{2}\right)a_{1}=-\alpha_{2}a_{1}^{2}a_{2}\cos\gamma_{1}-4f_{11}\cos\gamma_{2},\\ 8\omega_{3}a_{2}\left(3\sigma_{2}-\sigma_{1}\right)+\left(3\alpha_{8}a_{2}^{2}+2\alpha_{6}a_{1}^{2}\right)a_{2}=-\alpha_{5}a_{1}^{3}\cos\gamma_{1}. \end{array}\right.$$

An algebraic equation revealing the relationship between the vibration amplitude and other parameters can be derived by squaring the second and fourth formulas of Equation (47) and summing them as follows:

(48) $$\begin{array}{l} \left[32\omega_{3}\alpha_{6}a_{2}^{2}\left(3\sigma_{2}-\sigma_{1}\right)+12\alpha_{6}\alpha_{8}a_{2}^{4}\right]a_{1}^{2}+4\alpha_{6}^{2}a_{2}^{2}a_{1}^{4}-\alpha_{5}^{2}a_{1}^{6}\\ +9\alpha_{8}^{2}a_{2}^{6}+64\omega_{3}^{2}a_{2}^{2}\left[c_{3}^{2}+{\left(3\sigma_{2}-\sigma_{1}\right)}^{2}\right]+48\omega_{3}\alpha_{8}a_{2}^{4}\left(3\sigma_{2}-\sigma_{1}\right)=0. \end{array}$$

Letting $a_{1}^{2}=A_{1}$, Equation (48) can be simplified as a cubic equation for $A_{1}$,

(49) $$dA_{1}^{3}+eA_{1}^{2}+gA_{1}+h=0\left(d\neq 0\right),$$

where

(50) $$\begin{array}{l} d=\alpha_{5}^{2},\;e=-4\alpha_{6}^{2}a_{2}^{2},\\ g=-12\alpha_{6}\alpha_{8}a_{2}^{4}-32\omega_{3}\alpha_{6}a_{2}^{2}\left(3\sigma_{2}-\sigma_{1}\right),\\ h=-64\omega_{3}^{2}a_{2}^{2}\left[c_{3}^{2}+{\left(3\sigma_{2}-\sigma_{1}\right)}^{2}\right]-48\omega_{3}\alpha_{8}a_{2}^{4}\left(3\sigma_{2}-\sigma_{1}\right)-9\alpha_{8}^{2}a_{2}^{6}. \end{array}$$

Equation (49) can be rewritten as follows:

(51) $$x^{3}+px+q=0,$$

with $A_{1}=x-e/3d$, $p=\left(3dg-e^{2}\right)/3d^{2}$, and $q=\left(27d^{2}h\right.$$\left.-9deg+2e^{3}\right)/27d^{3}$. According to the Cardano formula, the solutions of Equation (51) are as follows:

(52) $$\left\{\begin{array}{l} x_{1}=\sqrt[{3}]{-\frac{q}{2}+\sqrt{{\left(\frac{q}{2}\right)}^{2}+{\left(\frac{p}{3}\right)}^{3}}}+\sqrt[{3}]{-\frac{q}{2}-\sqrt{{\left(\frac{q}{2}\right)}^{2}+{\left(\frac{p}{3}\right)}^{3}}},\\ x_{2}=\omega \sqrt[{3}]{-\frac{q}{2}+\sqrt{{\left(\frac{q}{2}\right)}^{2}+{\left(\frac{p}{3}\right)}^{3}}}+\omega^{2}\sqrt[{3}]{-\frac{q}{2}-\sqrt{{\left(\frac{q}{2}\right)}^{2}+{\left(\frac{p}{3}\right)}^{3}}},\\ x_{3}=\omega^{2}\sqrt[{3}]{-\frac{q}{2}+\sqrt{{\left(\frac{q}{2}\right)}^{2}+{\left(\frac{p}{3}\right)}^{3}}}+\omega \sqrt[{3}]{-\frac{q}{2}-\sqrt{{\left(\frac{q}{2}\right)}^{2}+{\left(\frac{p}{3}\right)}^{3}}}. \end{array}\right.$$

There is one real root and two complex roots in Equation (51) if $\Delta ≜{\left(q/2\right)}^{2}+{\left(p/3\right)}^{3}>0$. When Δ=0 and p,q≠0, there is one double root and one single root; the equation has three distinct real roots if Δ < 0.

$A_{1}$ is real because it is the vibration amplitude. Thus, we disregard the complex roots. Equation (48) provides the relationship between the low-frequency vibration amplitude $a_{1}$ and the high-frequency vibration amplitude $a_{2}$. Equation (52) implies that a given $a_{2}$ corresponds to one or three values of $a_{1}$. To simplify the research, we only consider that an $a_{2}$ corresponds to an $a_{1}$. The more intricate cases will be studied in another paper. Hence, we had

(53) $$A_{1}=\sqrt[{3}]{-\frac{q}{2}+\sqrt{{\left(\frac{q}{2}\right)}^{2}+{\left(\frac{p}{3}\right)}^{3}}}+\sqrt[{3}]{-\frac{q}{2}-\sqrt{{\left(\frac{q}{2}\right)}^{2}+{\left(\frac{p}{3}\right)}^{3}}}-\frac{e}{3d}.$$

Equation (53) can be transformed into

(54) $$\begin{array}{l} A_{1}={\left\{-\frac{27d^{2}h-9deg+2e^{3}}{54d^{3}}+{\left[{\left(\frac{27d^{2}h-9deg+2e^{3}}{54d^{3}}\right)}^{2}+{\left(\frac{3dg-e^{2}}{9d^{2}}\right)}^{3}\right]}^{1/2}\right\}}^{1/3}\\ +{\left\{-\frac{27d^{2}h-9deg+2e^{3}}{54d^{3}}+{\left[{\left(\frac{27d^{2}h-9deg+2e^{3}}{54d^{3}}\right)}^{2}+{\left(\frac{3dg-e^{2}}{9d^{2}}\right)}^{3}\right]}^{1/2}\right\}}^{1/3}-\frac{e}{3d}. \end{array}$$

From the second and the fourth formulas of Equation (46), we obtain

(55) $$\left\{\begin{array}{l} \sin\gamma_{1}=-8\omega_{3}c_{3}a_{2}/\alpha_{5}a_{1}^{3},\\ \cos\gamma_{1}=-\left[8\omega_{3}a_{2}\left(3\sigma_{2}-\sigma_{1}\right)+\left(3\alpha_{8}a_{2}^{2}+2\alpha_{6}a_{1}^{2}\right)a_{2}\right]/\alpha_{5}a_{1}^{3}. \end{array}\right.$$

Substituting $\sin\gamma_{1}$ and $\cos\gamma_{1}$ into the first and third formulas of Equation (47), and then squaring and summing the two equations, we obtain

(56) $$\begin{array}{c} 16f_{11}^{2}=64\omega_{1}^{2}a_{1}^{2}\left(c_{1}^{2}+\sigma_{2}^{2}\right)+{\left(8\omega_{3}c_{3}\alpha_{2}a_{2}^{2}/\alpha_{5}a_{1}\right)}^{2}+128\omega_{1}\omega_{3}c_{1}c_{3}\alpha_{2}a_{2}^{2}/\alpha_{5}\\ +{\left(3\alpha_{1}a_{1}^{3}+2\alpha_{3}a_{1}a_{2}^{2}\right)}^{2}+{\left[\left(8\omega_{3}\sigma_{1}\alpha_{2}a_{2}^{2}-24\omega_{3}\sigma_{2}\alpha_{2}a_{2}^{2}-3\alpha_{8}\alpha_{2}a_{2}^{4}-2\alpha_{6}\alpha_{2}a_{1}^{2}a_{2}^{2}\right)\right]}^{2}\\ +\omega_{1}\sigma_{2}\left(48\alpha_{1}a_{1}^{4}+32\alpha_{3}a_{1}^{2}a_{2}^{2}\right)+16\omega_{1}\alpha_{2}\sigma_{2}a_{2}^{2}\left[\left(8\omega_{3}\sigma_{1}-24\omega_{3}\sigma_{2}-3\alpha_{8}a_{2}^{2}-2\alpha_{6}a_{1}^{2}\right)/\alpha_{5}\right]\\ +\left(6\alpha_{1}\alpha_{2}a_{1}^{2}a_{2}^{2}+4\alpha_{2}\alpha_{3}a_{2}^{4}\right)\left[\left(-24\omega_{3}\sigma_{2}+8\omega_{3}\sigma_{1}-3\alpha_{8}a_{2}^{2}-2\alpha_{6}a_{1}^{2}\right)/\alpha_{5}\right] \end{array}$$

The vibration amplitude of the first- and third-order models can be obtained using the following procedure: First, $a_{1}^{2}$ is obtained using Equation (54); subsequently, $a_{1}^{2}$ is substituted into Equation (54) to find $a_{2}$. The angles $\gamma_{1}$ and $\gamma_{2}$ can be obtained by substituting $a_{1}$ and $a_{2}$ into the second and fourth formulas of Equation (47).

#### 2.2.4. Stability Analysis of Steady-State Solutions

The stability of the solutions can be determined by investigating the nature of the singular points in Equation (46). To accomplish this, we set $a_{1}=a_{1}^{0}+a_{1}^{1}$, $a_{2}=a_{2}^{0}+a_{2}^{1}$, $\gamma_{1}=\gamma_{1}^{0}+\gamma_{1}^{1}$, and $\gamma_{2}=\gamma_{2}^{0}+\gamma_{2}^{1}$. Subsequently, by substituting them into Equation (46) and considering that $\alpha_{j}^{0},\gamma_{j}^{0},j=1,2$ meet Equation (47), we obtain

(57) $$\left\{\begin{array}{c} {a^{\prime }}_{1}=\left(\alpha_{2}a_{1}^{2}a_{2}\sin\gamma_{1}+4f_{11}\sin\gamma_{2}\right)/8\omega_{1}-c_{1}a_{1}≜F_{1}\left(a_{1},a_{2},\gamma_{1},\gamma_{2}\right),\\ {a^{\prime }}_{2}=-\frac{\alpha_{5}a_{1}^{3}\sin\gamma_{1}}{8\omega_{3}}-c_{3}a_{2}≜F_{2}\left(a_{1},a_{2},\gamma_{1},\gamma_{2}\right),\\ {\gamma^{\prime }}_{2}=\left[\left(3\alpha_{1}a_{1}^{2}+2\alpha_{3}a_{2}^{2}\right)a_{1}+\alpha_{2}a_{1}^{2}a_{2}\cos\gamma_{1}+4f_{11}\cos\gamma_{2}\right]/8\omega_{1}a_{1}+\sigma_{2}≜F_{3}\left(a_{1},a_{2},\gamma_{1},\gamma_{2}\right),\\ {\gamma^{\prime }}_{1}=\left[\left(9\alpha_{1}a_{1}^{2}+6\alpha_{3}a_{2}^{2}\right)a_{1}+3\alpha_{2}a_{1}^{2}a_{2}\cos\gamma_{1}+12f_{11}\cos\gamma_{2}\right]/8\omega_{1}a_{1}\\ -\left[\left(3\alpha_{8}a_{2}^{2}+2\alpha_{6}a_{1}^{2}\right)a_{2}\right.\left.+\alpha_{5}a_{1}^{3}\cos\gamma_{1}\right]/8\omega_{3}a_{2}+\sigma_{1}≜F_{4}\left(a_{1},a_{2},\gamma_{1},\gamma_{2}\right). \end{array}\right.$$

The Jacobian matrix of Equation (57) is as follows:

(58) $$J=\left[\begin{array}{cccc} \frac{\partial F_{1}}{\partial a_{1}} & \frac{\partial F_{1}}{\partial a_{2}} & \frac{\partial F_{1}}{\partial \gamma_{2}} & \frac{\partial F_{1}}{\partial \gamma_{1}}\\ \frac{\partial F_{2}}{\partial a_{1}} & \frac{\partial F_{2}}{\partial a_{2}} & \frac{\partial F_{2}}{\partial \gamma_{2}} & \frac{\partial F_{2}}{\partial \gamma_{1}}\\ \frac{\partial F_{3}}{\partial a_{1}} & \frac{\partial F_{3}}{\partial a_{2}} & \frac{\partial F_{3}}{\partial \gamma_{2}} & \frac{\partial F_{3}}{\partial \gamma_{1}}\\ \frac{\partial F_{4}}{\partial a_{1}} & \frac{\partial F_{4}}{\partial a_{2}} & \frac{\partial F_{4}}{\partial \gamma_{2}} & \frac{\partial F_{4}}{\partial \gamma_{1}} \end{array}\right]$$

The elements in this matrix are shown in Appendix B. From Equation (47), we obtain

(59) $$\left\{\begin{array}{l} \gamma_{1}=\arctan\left[\frac{8\omega_{3}c_{3}}{8\omega_{3}\left(3\sigma_{2}-\sigma_{1}\right)+\left(3\alpha_{8}a_{2}^{2}+2\alpha_{6}a_{1}^{2}\right)}\right]\pm \pi ,\\ \gamma_{2}=\arctan\left\{-\left(8\alpha_{2}\omega_{3}c_{3}a_{2}^{2}+8\omega_{1}c_{1}\alpha_{5}a_{1}^{2}\right)\left\{\left[8\omega_{1}\sigma_{2}+\left(3\alpha_{1}a_{1}^{2}+2\alpha_{3}a_{2}^{2}\right)\right]\alpha_{5}a_{1}^{2}\right.\right.\\ \left.{\left.-\left[8\omega_{3}\left(3\sigma_{2}-\sigma_{1}\right)+\left(3\alpha_{8}a_{2}^{2}+2\alpha_{6}a_{1}^{2}\right)\right]\right\}}^{-1}\right\}\pm \pi \end{array}\right.$$

**Figure 8:**
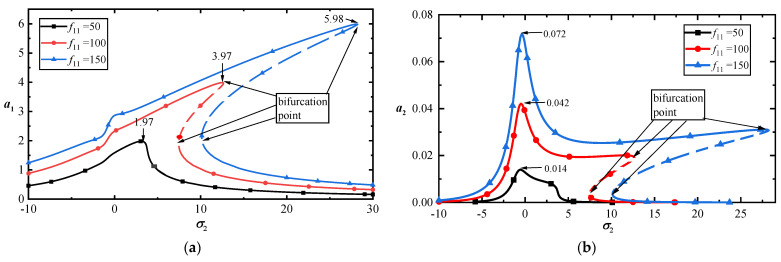
(**a**) Frequency–response curves of the first-mode vibration amplitudes with $\sigma_{2}$ for T=0; (**b**) frequency–response curves of the third-mode vibration amplitudes with $\sigma_{2}$ for T=0.

**Figure 9:**
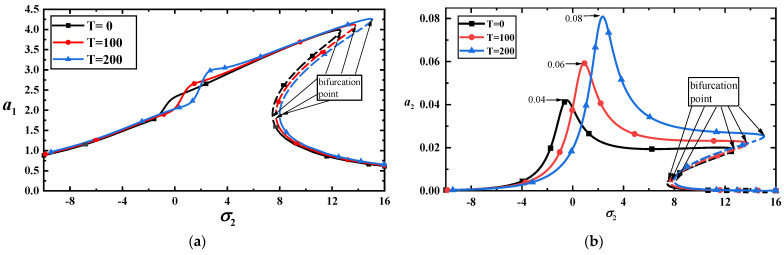
(**a**) Amplitude–frequency response curves of the first-mode vibration amplitudes with three temperatures for $f_{11}=100$; (**b**) amplitude–frequency response curves of the third-mode vibration amplitudes with three temperatures for $f_{11}=100$.

**Figure 10:**
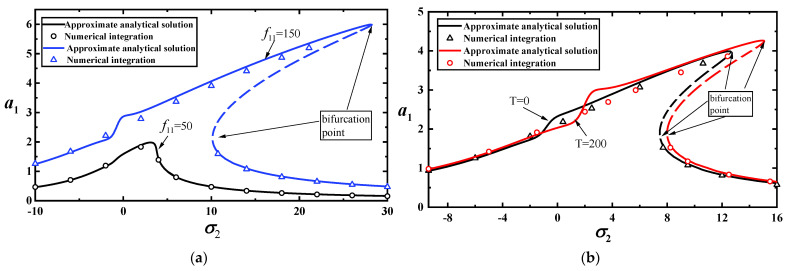
(**a**) Comparison between approximate analytical and numerical solutions for vibration amplitudes of the first-order model with two loads for T=0; (**b**) comparison between approximate analytical and numerical solutions for vibration amplitudes of the first-order model with two temperatures for $f_{11}=100$.

**Figure 11:**
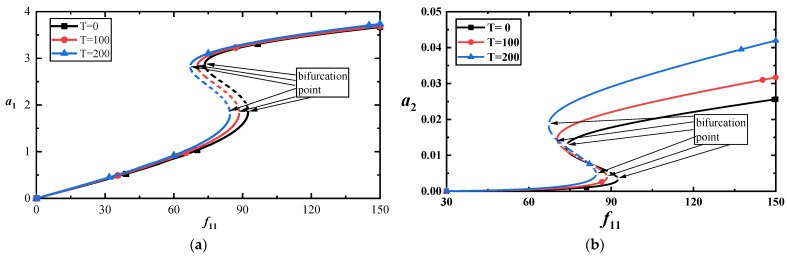
(**a**) Amplitude–response curves of the first-order mode for three temperatures at $\sigma_{2}=7$; (**b**) amplitude–response curves corresponding to the third-order mode for three temperatures at $\sigma_{2}=7$.

The stability of steady-state solutions can be determined using the eigenvalues of the Jacobian matrix J. The steady-state solution is unstable if the eigenvalues of the corresponding Jacobian matrix contain positive real components. The unstable solutions are indicated by the dotted lines in Figure 8, Figure 9, Figure 10 and Figure 11.

To study the 1:3 internal resonance, we use the mechanical and geometric coefficients listed in Table 1 and Table 3; the axial stresses are $N_{xx}^{0}=10\;\mathrm{nN}/\mathrm{nm},N_{yy}^{0}=49.8\;\mathrm{nN}/\mathrm{nm}$. By substituting these data into Equations (54) and (56), the relationship between the load frequency and amplitude can be displayed with a 1:3 internal resonance, as shown in Figure 8, Figure 9, Figure 10 and Figure 11.

Figure 8a,b illustrates the amplitude–frequency response curves of the first- and third-order modes with three external forces at T=0. The two figures show that the vibration amplitudes of the low-order mode are significantly larger than those of the high-order mode under the same loads. Because the nonlinear terms increase the stiffness of MoS_2_, the resonance peaks shift toward higher frequencies. Furthermore, the vibration amplitude may increase with frequency, which indicates significant changes in motion.

Figure 9a,b shows the effects of temperature on the amplitude–frequency response curves with $f_{11}=100$. The two figures reveal that the temperature has a more significant influence on the higher-order mode than on the lower-order mode. A slight temperature difference may induce an abrupt increase in the vibration amplitude. 

To validate the reliability of the approximate analytical solutions, we perform numerical calculations for Equation (20) using the Runge–Kutta method. A comparison between the analytical solution and numerical calculations is shown in Figure 10a (the first-order mode) and Figure 10b (the third-order mode). The results indicate that the approximate analytical solution is reliable.

**Figure 12:**
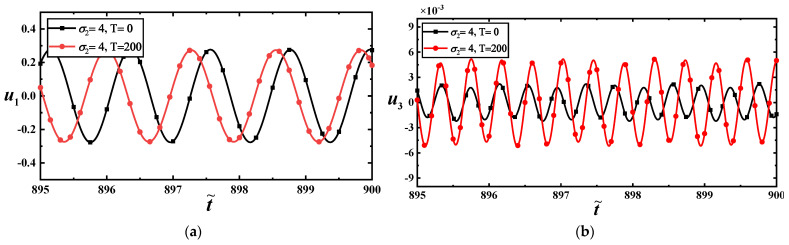
(**a**) Time–history response curves of the first-order mode with two temperatures at $f_{11}=100$; (**b**) time–history response curves of the third-order mode with two temperatures at $f_{11}=100$.

To show the effect of temperature on the vibration amplitude, we draw the load–amplitude response curves with $\sigma_{2}=7$ under different temperatures, as shown in Figure 11a,b. They imply that temperature has a more significant impact on the higher-order mode than on the low-order mode. The time–history curves display this feature, as shown in Figure 12a,b at $f_{11}=100$.

## 3. Conclusions

In this study, we employ a modified plate model in which four independent elastic parameters and thermal stresses are considered to investigate the nonlinear static bending and vibrations of monolayer MoS_2_. First, we use the Galerkin method to truncate the partial differential equation with the first and third modes. Subsequently, nonlinear static bending and forced vibrations are explored using ordinary differential equations obtained using the Galerkin method. The main conclusions are as follows:

(1) The first-order mode can accurately represent the static deformation of MoS_2_ under symmetric loads.
(2) Temperature has a slight effect on the single-mode vibrations of the MoS_2_. However, the combination of the load frequency and temperature have a more significant effect on the vibrations. When the temperature has a slight change, the bifurcation points of vibration amplitude will change significantly with the identical load’s amplitude and frequency.
(3) The bifurcation points of the load at the low-frequency primary resonance are significantly smaller than those at the high frequency for single-mode vibrations.
(4) The vibration amplitudes of the first-order mode are significantly larger than those of the higher-order modes under the same loads when a 1:3 internal resonance appear in the MoS_2_.
(5) For the 1:3 internal resonance, the temperature has a more significant influence on the higher-order mode than on the lower-order mode, and a slight temperature difference may induce an unexpected jump in the vibration amplitude. Under the same load, the maximum value of the amplitude–frequency curve will increase significantly with the temperature’s increase.

The above findings may give some important inspirations when a single-layer MoS_2_ is used in nano-resonators and mass sensors.

## Figures and Tables

**Table 1:** Mechanical parameters of the single-layered MoS_2_ [28,37,38].

$\kappa_{B}\left(eV\right)$	$Y\left(N/m\right)$	V	$\alpha \left(K^{-1}\right)$	$\kappa_{b}\left(eV/nm^{2}\right)$	$\kappa_{g}\left(eV/nm^{2}\right)$	${\hat{c}}_{j}$
9.61	120	0.23	6.49 × 10^−5^	792	610	0.05

**Table 2:** The partial mechanical parameters of the classical Föppl-von Karman plate model for $a=5,b=10,N_{xx}^{T}=0.3,N_{yy}^{T}=0.1,T=50.$

$h\left(nm\right)$	${Κ}_{B}\left(eV\right)$	$\omega_{1}$	$\omega_{3}$
0.445	13.05	1.1544	9.1788
0.65	27.85	1.2062	9.2167

**Table 3:** Coefficients in Equation (56) for $a=5,b=15,N_{xx}^{T}=10,N_{yy}^{T}=49.8$.

T	First-Order Natural Frequency	Third-Order Natural Frequency	$\mathrm{Detuning}\;\mathrm{Parameter}\;\sigma_{1}$
0	$\omega_{1}$ = 5.18	$\omega_{3}$ = 15.50	−4
100	$\omega_{1}$ = 5.03	$\omega_{3}$ = 15.09	0.914
200	$\omega_{1}$ = 4.88	$\omega_{3}$ = 14.68	4

## Data Availability

No new data were created.

## Appendix A

The parameters of Equation (20):

$$\begin{array}{c} \omega_{1}^{2}=\frac{{\left(b^{2}+a^{2}\right)}^{2}}{b^{4}}+\frac{a^{2}(N_{xx}^{0}-N_{xx}^{T})}{K_{B}\pi^{2}}+\frac{a^{4}(N_{yy}^{0}-N_{yy}^{T})}{b^{2}K_{B}\pi^{2}},\;\\ f_{1}=\frac{16a^{3}f}{\pi^{6}K_{B}},\;f_{3}=\frac{16a^{3}f}{3K_{B}\pi^{6}},\;\\ \omega_{2}^{2}=\frac{{\left(9b^{2}+a^{2}\right)}^{2}}{b^{4}}+\frac{9a^{2}(N_{xx}^{0}-N_{xx}^{T})}{K_{B}\pi^{2}}+\frac{a^{4}(N_{yy}^{0}-N_{yy}^{T})}{b^{2}K_{B}\pi^{2}},\\ \alpha_{1}=-\left[\frac{56.89a^{6}\chi }{K_{B}\pi^{4}{\left(a^{2}+b^{2}\right)}^{2}}+\frac{55.62a^{6}\chi }{K_{B}\pi^{4}{\left(a^{2}+9b^{2}\right)}^{2}}\right],\;\\ \alpha_{2}=\left[\frac{125.16a^{6}\chi }{K_{B}\pi^{4}{\left(a^{2}+b^{2}\right)}^{2}}+\frac{1029.34a^{6}\chi }{K_{B}\pi^{4}{\left(a^{2}+9b^{2}\right)}^{2}}\right],\\ \alpha_{3}=-\left[\frac{339.13a^{6}\chi }{K_{B}\pi^{4}{\left(a^{2}+b^{2}\right)}^{2}}\right.\left.+\frac{4918.36a^{6}\chi }{K_{B}\pi^{4}{\left(a^{2}+9b^{2}\right)}^{2}}\right],\;\\ \alpha_{4}=\left[\frac{203.82a^{6}\chi }{K_{B}\pi^{4}{\left(a^{2}+b^{2}\right)}^{2}}-\frac{1579.89a^{6}\chi }{K_{B}\pi^{4}{\left(a^{2}+9b^{2}\right)}^{2}}\right],\\ \alpha_{5}=\frac{41.72a^{6}\chi }{K_{B}\pi^{4}{\left(a^{2}+b^{2}\right)}^{2}}+\frac{214.55a^{6}\chi }{K_{B}\pi^{4}{\left(a^{2}+9b^{2}\right)}^{2}},\;\\ \alpha_{6}=-\left[\frac{339.13a^{6}\chi }{K_{B}\pi^{4}{\left(a^{2}+b^{2}\right)}^{2}}\right.+\left.\frac{2691.97a^{6}\chi }{K_{B}\pi^{4}{\left(a^{2}+9b^{2}\right)}^{2}}\right],\\ \alpha_{7}=\left[\frac{611.47a^{6}\chi }{K_{B}\pi^{4}{\left(a^{2}+b^{2}\right)}^{2}}\right.-\left.\frac{3101.26a^{6}\chi }{K_{B}\pi^{4}{\left(a^{2}+9b^{2}\right)}^{2}}\right],\\ \alpha_{8}=-\left[\frac{1357.95a^{6}\chi }{K_{B}\pi^{4}{\left(a^{2}+b^{2}\right)}^{2}}+\frac{682.7a^{6}\chi }{K_{B}\pi^{4}{\left(a^{2}+9b^{2}\right)}^{2}}\right]. \end{array}$$

## Appendix B

The Jacobian matrix coefficients of Equation (58) are as follows:

$$\begin{array}{c} \frac{\partial F_{1}}{\partial a_{1}}=\frac{\alpha_{2}a_{1}a_{2}\sin\gamma_{1}}{4\omega_{1}}-c_{1},\frac{\partial F_{1}}{\partial a_{2}}=\frac{\alpha_{2}a_{1}^{2}\sin\gamma_{1}}{8\omega_{1}},\frac{\partial F_{1}}{\partial \gamma_{2}}=\frac{f_{11}\cos\gamma_{2}}{2\omega_{1}},\\ \frac{\partial F_{1}}{\partial \gamma_{1}}=\frac{\alpha_{2}a_{1}^{2}a_{2}\cos\gamma_{1}}{8\omega_{1}},\frac{\partial F_{2}}{\partial a_{1}}=-\frac{\alpha_{5}3a_{1}^{2}\sin\gamma_{1}}{8\omega_{3}},\frac{\partial F_{2}}{\partial a_{2}}=-c_{3},\\ \frac{\partial F_{2}}{\partial \gamma_{2}}=0,\frac{\partial F_{2}}{\partial \gamma_{1}}=-\frac{\alpha_{5}a_{1}^{3}\cos\gamma_{1}}{8\omega_{3}},\\ \frac{\partial F_{3}}{\partial a_{1}}=\frac{6\alpha_{1}a_{1}+\alpha_{2}a_{2}\cos\gamma_{1}}{8\omega_{1}}-\frac{f_{11}\cos\gamma_{2}}{2\omega_{1}a_{1}^{2}},\\ \frac{\partial F_{3}}{\partial a_{2}}=\frac{4\alpha_{3}a_{2}a_{1}+\alpha_{2}a_{1}^{2}\cos\gamma_{1}}{8\omega_{1}a_{1}},\frac{\partial F_{3}}{\partial \gamma_{2}}=-\frac{f_{11}\sin\gamma_{2}}{2\omega_{1}a_{1}},\\ \frac{\partial F_{3}}{\partial \gamma_{1}}=-\frac{\alpha_{2}a_{1}^{2}a_{2}\sin\gamma_{1}}{8\omega_{1}a_{1}},\\ \frac{\partial F_{4}}{\partial a_{1}}=\frac{18\alpha_{1}a_{1}+3\alpha_{2}a_{2}\cos\gamma_{1}}{8\omega_{1}}-\frac{3f_{11}\cos\gamma_{2}}{2\omega_{1}a_{1}^{2}}-\frac{4\alpha_{6}a_{1}a_{2}+3\alpha_{5}a_{1}^{2}\cos\gamma_{1}}{8\omega_{3}a_{2}},\\ \frac{\partial F_{4}}{\partial a_{2}}=\frac{12\alpha_{3}a_{2}+3\alpha_{2}a_{1}\cos\gamma_{1}}{8\omega_{1}}-\frac{3\alpha_{8}a_{2}}{4\omega_{3}}+\frac{\alpha_{5}a_{1}^{3}\cos\gamma_{1}}{8\omega_{3}a_{2}^{2}},\\ \frac{\partial F_{4}}{\partial \gamma_{2}}=-\frac{3f_{11}\sin\gamma_{2}}{2\omega_{1}a_{1}},\frac{\partial F_{4}}{\partial \gamma_{1}}=\frac{\alpha_{5}a_{1}^{3}\sin\gamma_{1}}{8\omega_{3}a_{2}}-\frac{3\alpha_{2}a_{1}^{2}a_{2}\sin\gamma_{1}}{8\omega_{1}a_{1}}. \end{array}$$
